# Engagement of atrial fibrillation patients with the AF-EduApp, a new mobile application to support AF management

**DOI:** 10.3389/fcvm.2023.1243783

**Published:** 2023-09-26

**Authors:** Lieselotte Knaepen, Michiel Delesie, Rik Theunis, Peter Gorissen, Johan Vijgen, Paul Dendale, Lien Desteghe, Hein Heidbuchel

**Affiliations:** ^1^ Department of Cardiology, Antwerp University Hospital, Edegem, Belgium; ^2^ Research Group Cardiovascular Diseases, University of Antwerp, Antwerp, Belgium; ^3^ Faculty of Medicine and Life Sciences, Uhasselt, Diepenbeek, Belgium; ^4^ Department of Cardiology, Heart Centre Hasselt and Department Jessa & Science, Jessa Hospital, LCRC(-MHU), Hasselt, Belgium

**Keywords:** atrial fibrillation, mHealth, mobile application, integrated care, education

## Abstract

**Introduction:**

A multidisciplinary approach is needed for the management of atrial fibrillation (AF) in which the patient has a central role. Smart devices create opportunities to improve AF management. This paper aimed to evaluate the in-house developed AF-EduApp application on its usability, satisfaction, and communication effectiveness with the care team.

**Methods:**

During a multicenter, prospective randomized controlled trial, 153 AF patients were included in the AF-EduApp study, with a minimum follow-up of 12 months and a maximum follow-up of 15 months if taking oral anticoagulation (OAC). The AF-EduApp contains six main modules: Questionnaires, Education, Measurement data entry, Medication overview with reminders, Appointments, and Communication with the care team. The App focuses on four main goals: (1) to improve AF knowledge, (2) to increase self-care capabilities, (3) electronic monitoring to improve therapy adherence to OAC, and (4) communication with the care team. Patients unable to use the AF-EduApp were assigned to a no-App control group (*n* = 41) without intervention comparable to the standard care group (SC, *n* = 346) of the AF-EduCare study.

**Results:**

A total of 152 patients effectively used the App during a mean follow-up of 386.8 ± 108. 1 days (one included patient could not install the application due to an iPhone from the United States). They opened the application on average on 130.1 ± 144.7 days. Of the 109 patients still in follow-up after 12 months (i.e. patients who did not withdraw and on OAC), 90 patients (82.6%) actively used the application at least one day in the next 41 days. The Measurement module was the most used, with a median of used days over the total available days of 6.4%. A total of 75 App patients (49.3%) asked questions, mostly clinical-related questions (e.g. medication use, or actionability on clinical entered parameters). A mean score of 8.1 ± 1.7 about the “perceived quality of follow-up in the past year” was given by the App ITT patients, compared to a score of 7.7 ± 2.0 by the SC group (*P* = .072). Patients who used the App were more attracted to future follow-up with an application compared to patients who would be capable of using the application of the SC group (31.6% vs. 12.5%; *P* < .001).

**Conclusion:**

This study showed a positive attitude towards using a mobile application, with AF patients using the application one-third of the available days. Patients used the App most for entering measured parameters, and to contact the care team.

## 1. Introduction

With a lifetime risk of one in three, AF is the most prevalent cardiac arrhythmia, affecting 59 million people globally (1, 2). This arrhythmia is associated with increased morbidity and mortality, including potentially life-threatening complications such as stroke or heart failure (3). The management of AF is based on three pillars, also called the ABC pathway: (A) avoiding stroke with anticoagulation treatment, (B) better symptom control based on rate and rhythm control strategies, and (C) cardiovascular risk factor management. A recently published study showed that following the ABC approach was linked to greater health-related quality of life and a significantly decreased risk of the composite outcome of all-cause death and any thromboembolism in secondary prevention AF patients (4). This was also confirmed by the review of Stevens et al., which showed that patients receiving care following the ABC pathway had significantly lower risks of stroke, myocardial infarction, and death (5). However, recent studies have revealed that the ABC strategy is still not being followed to its full potential. More particularly, around 1 in 5 patients do not receive ABC-based care (6–8). As the ABC pathway shows, the management of AF is complex and needs to be patient-tailored. Accordingly, patient education and involvement are crucial to allow shared-decision making. This shared-decision making is only achievable if patients with AF are adequately educated about their condition and its management. Education as the entry point to involvement in their personal care was the foundation of a large, multicenter, randomized controlled trial, the AF-EduCare study. This study compares in-person and online-driven educational follow-ups to standard care (9). The current study is a derivative of this larger trial.

In the past decades, smartphones have become more popular and indispensable devices for the general population. Even in the 65 + age population, smartphones became the second most important device after a portable computer (PC) (10). In Belgium, smartphone ownership increased from 48% in 2017 to 82% in 2021 in this population, which is comparable with other countries and age populations (10–12). Such devices allow for a remote follow-up of patients, supplementing other forms of care, hence creating more continuous care. Besides, they can be ideal tools to educate patients on different aspects of their condition. This digital growth led to the in-house development of the AF-EduApp (13). This allowed us to follow up with AF patients with more opportunities and functionalities compared to online follow-up. Given the similar follow-up strategy to the AF-EduCare study, the AF-EduApp study (NCT03788044) was added as a fourth study arm to the AF-EduCare study. The primary outcome parameter of the AF-EduApp study was to improve therapy adherence to oral anticoagulation (OAC) by using this educational smartphone application with an integrated medication list and reminders. The results showed that patients who installed a medication reminder had a higher therapy adherence to OAC than those patients who used the app but did not install a medication reminder (14). Other outcome parameters such as AF knowledge, self-care capabilities, AF symptom burden and quality of life are expected in the first half of 2024. The current report focuses on patients' satisfaction with the application and the usability of the application-driven follow-up strategy.

## 2. Methods

### 2.1. AF-EduApp development and design

A prototype of the AF-EduApp application was designed, developed, and optimized before the start of the AF-EduApp study (13). The validation process consisted of three parts: (1) an expert panel gave feedback about the content and functionality; (2) ten AF patients expressed orally what they thought of the application while using it in the presence of a researcher; (3) 20 AF patients used the application during a pilot study of one month.

Next, the in-house developed and optimized AF-EduApp application was made available for smartphones and tablets on IOS or Android for the AF-EduApp study. Every patient had a unique log-in and received access to the six different modules **(**Supplementary Figure S1**)**: i.e., (i) an Education module about AF and its treatment which was available 24/7 (except when the knowledge questionnaire had to be completed); (ii) a Questionnaire module including various questionnaires that patients were requested to complete during the study; (iii) a module in which patients could record their medication list, and had the possibility to install an alarm when they had to take a specific medication. They also could record whether they had taken the medication; (iv) a module to enter measured parameters such as weight and blood pressure; (v) a module where upcoming appointments could be inserted and (vi) lastly a module with contact information and a chat box in case of questions for the care team. Of note, the patients had no predefined instructions on how often and how to use the App. They were free to decide how frequently they wanted to use it (except for the questionnaires that needed to be completed within specific time frames and were presented automatically).

### 2.2. Study design

The AF-EduApp study (NCT03788044) is incorporated as a fourth study arm in the larger multicenter, randomized AF-EduCare trial (NCT03707873) (9). In this AF-EduCare study, the effectiveness of several individualized education initiatives (i.e., in-person, online, and App-based education) is compared to conventional care regarding clinical outcome metrics for AF patients. In short, the intervention's (AF-EduCare/AF-EduApp) primary focus is improving patients' knowledge about AF and its treatment. Based on this improved knowledge, two other aspects of AF treatment are targeted: promoting self-care skills based on individual risk factors for AF and increasing adherence to OAC therapy. The easy reachability of the AF team was also part of the AF-EduCare/AF-EduApp approach. Patients were randomly assigned to the four study groups: in-person education, online education, App-driven education, and standard care (SC).

Both trials were approved by the Ethical committees of the participating centers, and the studies have been conducted in compliance with the Declaration of Helsinki. More details on the AF-EduCare study design have already been described by *Delesie et al*. (9). In addition, baseline demographics of the screened AF population (*n* = 1.979) for the AF-EduCare/AF-EduApp have been described, including a CONSORT diagram and more details on the 1.232 included AF patients (15). As this analysis focuses on the AF-EduApp group, the study protocol of the AF-EduApp study arm will be explained in more detail below. The focus of this report is the methods used to assess patient satisfaction, communication, and App engagement. The SC group (*n* = 346) was used to compare patient satisfaction with the AF-EduApp study arm (*n* = 194). This SC group had a comparable clinical profile due to stratification with the randomization procedure.

### 2.3. Study population and randomization

We aimed to include 153 AF App eligible patients (hospitalized or outpatient at two hospitals, University Hospital Antwerp and Jessa Hospital Hasselt). The inclusion criteria were: ≥18 years, AF or atrial flutter diagnosed with an electrocardiogram, and the capability to sign the informed consent. Patients were excluded if they could not speak Dutch, were cognitively impaired, had a life expectancy estimated to be less than a year, were pregnant, or participated in other clinical trials. There was one randomization procedure for both the AF-EduCare and AF-EduApp studies. Possibility to work with a smartphone application was not used as an inclusion criterion in order to perform both on treatment and intention to treat analyses. Therefore, patients randomly assigned to the App-driven education group could be divided into those who can (eligible; App group) and cannot (non-eligible; no-App group) follow App-driven education, i.e., based on the availability of a smartphone/tablet and the ability of the patient to use it. Patients unable to pursue the App-driven education were followed as a control care group (non-eligible group, Supplementary Figure S2) in addition to the SC group.

### 2.4. Data collection and recruitment

Patients in the AF-EduApp study arm had a follow-up of 12 to 15 months (depending on an extra period with therapy adherence monitoring). Basic demographic data of all patients were assessed at baseline. Different outcome parameters were evaluated, as shown in Supplementary Figure S2. Patients in the no-App group had to complete the questionnaires with a tablet at the hospital. Four parameters of the intervention were analyzed; (I) patients' knowledge about AF and its treatment was evaluated with the Jessa Atrial fibrillation Knowledge Questionnaire (JAKQ) with targeted education based on wrongly answered questions (16), (II) self-care capabilities using the self-developed self-care questionnaire (SCQ), (III) therapy adherence to OAC was monitored using the electronic Medication Events Monitoring System (MEMS) and based on confirmation of medication intake with the alarms, and lastly (IV) during the entire follow-up, patients of the on-treatment App group were able to contact the study personnel during regular working hours if they had questions about AF or its management. Patients of both intervention and control groups received a general information brochure about AF with contact information. Additional outcomes were assessed, like quality of life, symptom burden, and intervention satisfaction, according to the AF-EduCare study design (9). This report focuses only on App's usability, questions raised by App patients, and satisfaction with the App intervention compared to SC. For the latter one, intention-to-treat analyses with App and no-App groups together (App ITT) were performed compared to SC. In addition, to assess the effect of being capable to use an application and effectively using it, App-capable and App non-capable patients were compared.

#### 2.4.1. AF-EduApp engagement measurements

In the patients using the App, the AF-EduApp kept track of how long and how often patients used the different modules. Every click a patient made on the application was registered in a database. In this way, it was possible to investigate the engagement of patients with the App and which modules were most popular. App user data were described until 12 or 15 months, depending on whether patients had therapy adherence monitoring or not. Therapy adherence monitoring was performed with AF patients treated with an OAC at baseline or initiated during the study. These patients were monitored with the MEMS cap at the beginning of the study (Monitoring 1, M1) and again after one year (Monitoring 2, M2), each time for a period of three months. The MEMS cap fits on a medication bottle, and each time the bottle is opened, data is stored in the MEMS cap build-in memory card. The study team extracted the data from the cap at the 3- and 15-months follow-up visits. Regimen adherence was calculated as the proportion of days with the correct number of openings as recorded by MEMS, once or twice daily, depending on the dosing regimen.

#### 2.4.2. Communication measurements

During the study, patients could contact the care team (i.e., mostly nurses trained in AF management) by phone or via the application to ask questions about AF and its management. If necessary for clinical related questions, the study personnel consulted the treating physician before responding and could implement an action plan if needed. The specific questions asked by the patients and the time necessary to answer these questions and take action were tracked and recorded in the eCRF.

#### 2.4.3. Satisfaction of the AF-EduApp intervention

After 12 or 18 months, patients were asked about their opinion on the educational efforts given during the study project (by the AF study team in the App group or by physicians as part of standard care in the no-App and SC groups). Patient satisfaction was assessed through a study-specific Patient Reported Outcome and Experience Measures (PROM & PREM) questionnaire comprising three main domains. Patients in the App and no-App or SC groups received 20 and five questions, respectively. The first questions are about general health after the study and how the patients would like to be informed about their rhythm disorder. These were followed by intervention-specific multiple-choice questions on the educational efforts and the application. In the end, patients could give a final score on a scale from one to ten regarding general education. They were given the opportunity to suggest how future education could be further optimized.

### 2.5. Statistical analysis

The sample size calculation was based on the primary endpoint of regimen adherence to OAC medication after 12 months. This calculation included improved therapy adherence from 89.63% ± 14.48% (17) to 96.84% by the application-driven intervention. Considering that 64.4% of the AF population is on OAC (16), a sample size of 153 patients was calculated who had to use the application. The statistical analyses were performed using SPSS 28.0. (IBM, Armonk, USA). Normal distribution was assessed with the Shapiro-Wilk test. As appropriate, variables were described as numbers and percentages, as mean ± standard deviation (SD) or median and interquartile range (IQR). For continuous variables, the Mann-Withney U test was used to compare two groups and the Kruskal Wallis for two or more categories. The chi-squared test was used for categorical variables. *P*-values <0.05 were considered statistically significant. Because patients were followed up over time and user data was analyzed, participation statuses (yes/no) obtained from the same patient were expected to be correlated. Ignoring correlation would typically result in underestimating standard errors and hence wrong conclusions (18). Therefore, a generalized estimating equation (GEE) model was constructed using participation as a binary outcome, a logit link function, and an autoregressive working correlation (19).

## 3. Results

A total of 194 AF patients (App: 153; no-App: 41) were included in the AF-EduApp study arm. The patients had a mean follow-up of 12.8 ± 3.4 months. A total of 25 patients (12.9%; App 19 (12.4%), and six no-App (14.6%)) did not complete the entire follow-up due to different reasons **(**Supplementary Figure S3**)**. As expected, App and no-App patients differed significantly in many respects: the no-App group was significantly older (77.9 ± 5.5 and 70.7 ± 6.6, respectively; *P* < .001), had a higher CHA_2_DS_2_-VASc score (4.2 ± 1.7 and 2.9 ± 1.4, respectively; *P* < .001), and had less access to digital devices, as shown in Supplementary Table S1. For this reason, satisfaction with the intervention was compared between the intention-to-treat App group (App ITT, *n* = 194) and SC (*n* = 346). As shown in Table 1, these groups were well-matched.

**Table 1:** Demographic data of included cardiology patients.

	Total population (*n *= 540)	AF-EduApp (ITT) (*n *= 194)	SC (*n *= 346)	*p*-value^a^
Male, *n* (%)	372 (68.9)	132 (68.0)	240 (69.4)	.75
Age (years), mean ± SD	70.0 ± 8.4	70.1 ± 7.0	69.9 ± 9.1	.784
Education degree, *n* (%)				.61
Primary /secondary school	314 (58.1)	110 (56.7)	204 (59.0)	
College/University	226 (41.9)	84 (43.3)	142 (41.0)	
In possession of a device, *n* (%)	480 (88.9)	179 (92.3)	301 (87.0)	.061
PC/Laptop	438 (81.1)	154 (79.4)	284 (82.1)	.442
Tablet	257 (47.6)	105 (54.1)	152 (43.9)	**.023**
Smartphone	350 (64.8)	156 (80.4)	194 (56.1)	<**.001**
Internet accessibility, *n* (%)	485 (89.8)	181 (93.3)	304 (87.9)	**.045**
Time since AF diagnosis (years), mean ± SD	5.8 ± 7.1	5.1 ± 6.4	6.2 ± 7.5	.151
CHA2DS2-VASc score, mean ± SD	3.1 ± 1.7	3.1 ± 1.6	3.1 ± 1.7	.763
Anticoagulation therapy				.312
NOAC	440 (84.0)	163 (84.0)	277 (80.1)	
VKA	47 (8.7)	16 (8.2)	31 (9.0)	
LMWH	5 (0.9)	0 (0.0)	5 (1.4)	
None	48 (8.9)	15 (7.7)	33 (9.5)	

Bold values are significant *p*-values (i.e., *p* < 0.05).

^a^ A Mann-Whitney U test was used for continuous data and a Chi-square test was used for categorical data.

AF, atrial fibrillation; NOAC, Non-vitamin K antagonist Oral Anticoagulant; VKA, Vitamin K Antagonist; LMWH, Low-Molecular-Weight Heparins.

### 3.1. AF-EduApp engagement

A total of 152 patients started to use the AF-EduApp (one included patient could not install the application due to an iPhone from the United States) with, on average, 386.8 ± 108.1 follow-up days and 130.1 ± 144.7 days of usage of at least one module of the application (i.e., 33.6% of the days).

Patients with an AF diagnosis <1 month before inclusion used the application significantly less (*n* = 35; 66.8 ± 89.6 days) compared with patients who had their AF diagnosis >1 month before inclusion (*n* = 117; 149.0 ± 152.8 days) (*P* = .003). It has to be taken into account that patients with an AF diagnosis <1 month before inclusion had significantly lower follow-up days (312.9 ± 163.2) compared to patients with a longer time of AF diagnosis (409.0 ± 72.6; *P* = .002). This difference was also seen for the drop-out rate before 12 months (<1 month: 13 (36.2%) vs. >1 month: 10 (8.5%); *P* < .001), with most patients with recent AF diagnosis had a drop-out already after one-month follow-up [*n* = 10 (27.8%)]. Despite correction for follow-up days, patients with recent AF diagnosis spent a significantly lower number of days in the Education module (AF diagnosis <1 month: 0.22 (0–0.89) % days vs. AF diagnosis >1 month: 0.67 (0.27–1.33) % days; *P* = .002), while they used the Questionnaire module more frequently (1.44 (1.12–2.22) % days vs. 1.32 (1.11–1.57) % days respectively; *P* = .023). However, no significant difference in knowledge based on the JAKQ was seen at baseline (69.6 ± 16.9% vs. 69.8 ± 14.6%; *P* = .920).

**Figure 1:**
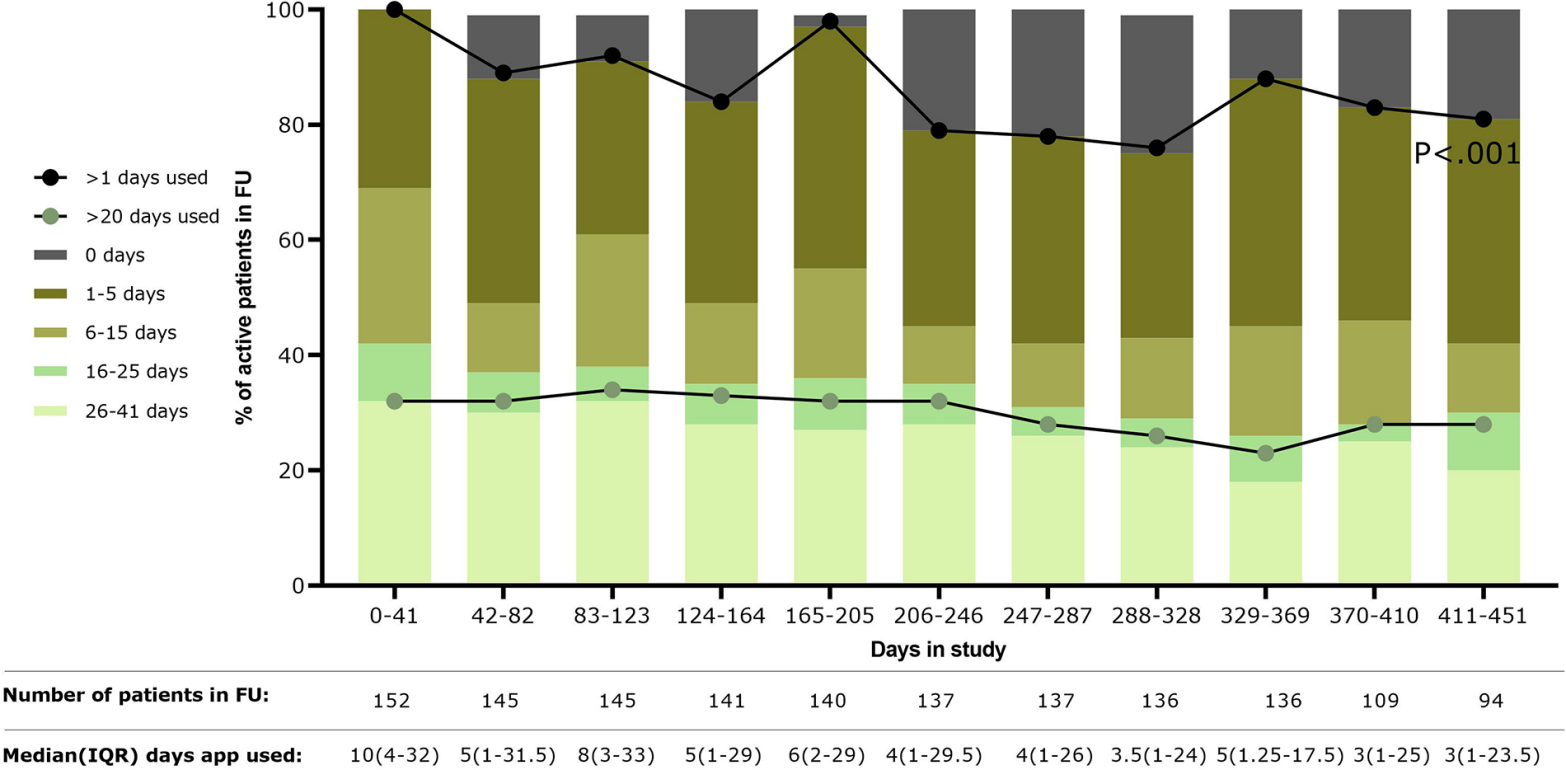
Usage of the application over time is shown as the percentage of the number of patients using the application over the total patients still in follow-up during that time period (shown in table below the figure). In addition, median days of using the application by the active patients was shown in the table below the figure. FU, follow-up; IQR, interquartile range.

The number of patients using the application over the total active patients (i.e., patients still in follow-up at a certain point in time) decreased over time **(**Figure 1**)**. Most patients used the application between 1 and 15 days in sub-periods of 41 days, with the median number of days used per period progressively decreasing over time (*P* < .001). An increase in the number of patients using the application was seen during periods in which questionnaires were automatically presented (i.e., during the following four periods: 0–41 days, 82–123 days, 164–205 days, and 328–369 days). However, at the end of the study (410–451 days), 81% (76/94 patients) of the active patients used the application at least once.

**Figure 2:**
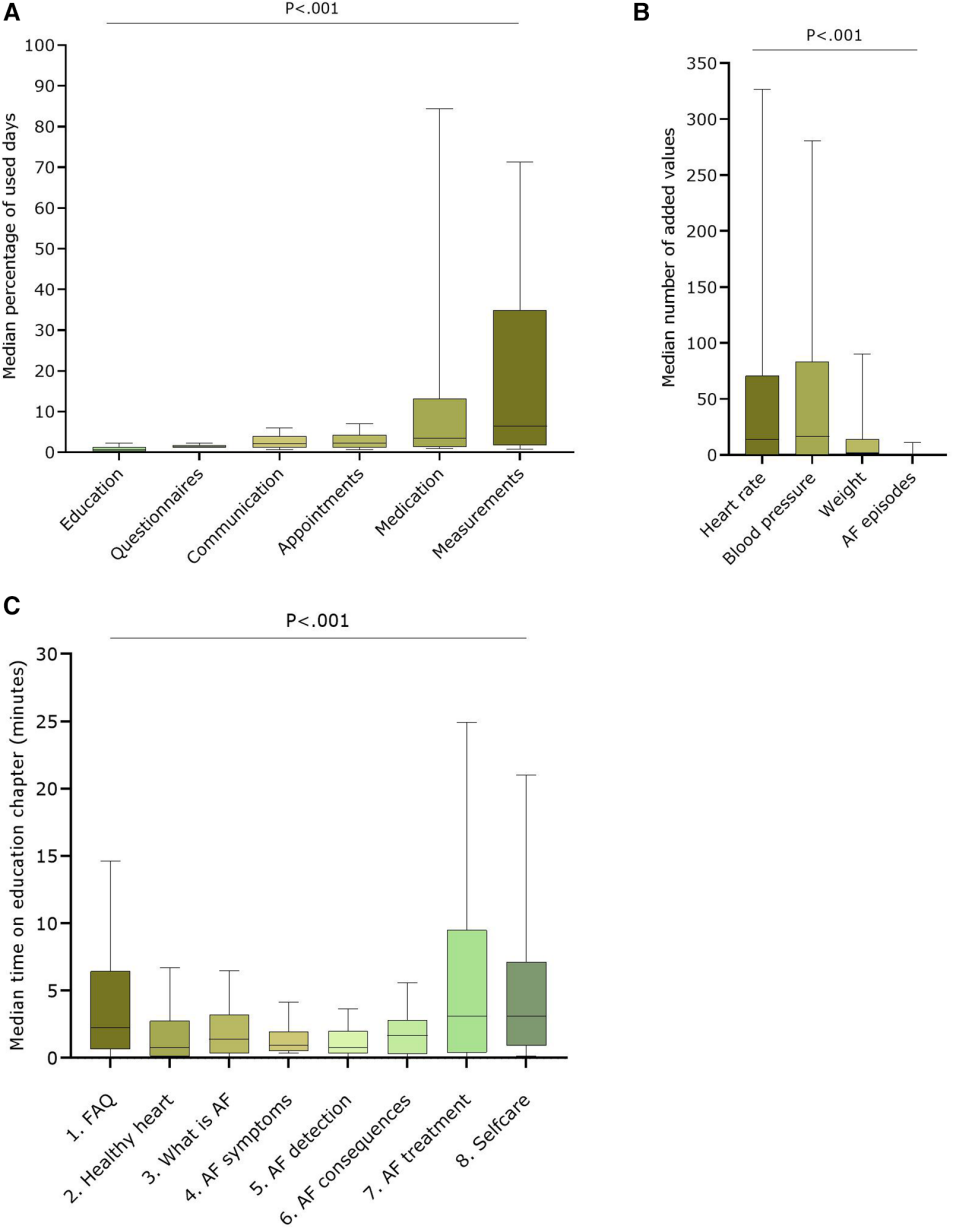
User data of the different modules over the entire study period (**A**) Median percentage of used days over total follow-up days for the six available modules in the AF-EduApp (*n* = 152); (**B**) Median number of added parameters in the measurement module (*n* = 152); (**C**) Median time spent on each chapter of the education module (*n* = 135). AF, atrial fibrillation; Adh, therapy adherence, FAQ, Frequently Asked Questions. Part A, B and D are shown as boxplots with median, IQR and 10-90 percentiles.

Almost all patients viewed most modules at least once (i.e.,: Questionnaires: 152; Medication: 151; Measurements: 150; Communication: 150; Appointments: 149; Education: 135). A median percentage was calculated based on how many days a patient used a module over the total number of follow-up days of that patient (Figure 2A**)**. Based on these results, the Measurement and Medication modules were most used, respectively 6.4 (1.8–30.7)% and 3.3 (1.4–10.9)% days over an average of 386.8 ± 108.1 follow-up days. The Education module was used the least [median 0.8 (0.2–1.3)% of the available days], despite (or because of) the fact that the application automatically presented questionnaires at regular intervals. Within the Measurement module, heart rate and blood pressure were the most entered parameters (median of 14.0 (0.0–70.8) and 16.5 (0.0–83.3) entries, respectively per patient) **(**Figure 2B**)**. The Education module was the least visited module, with 135 patients visiting at least once a specific chapter. The median time spent on the Education module was 3.1 (1.3–21.8) minutes during the whole study (mean: 18.6 ± 32.9 min). There was no significant difference between patients with a recent AF diagnosis (<1 month) [*n* = 18; median: 8.8 (3.4–21.8) minutes] and patients who had a diagnosis longer than one month before inclusion (*n* = 117; median: 5.8 (1.1–21.6 min) (*P* = .370). As shown in Figure 2C, most time was spent on the chapters about AF treatment (3.1 (0.4–9.5) minutes) and self-care [3.1 (0.9–7.1) minutes].

**Figure 3:**
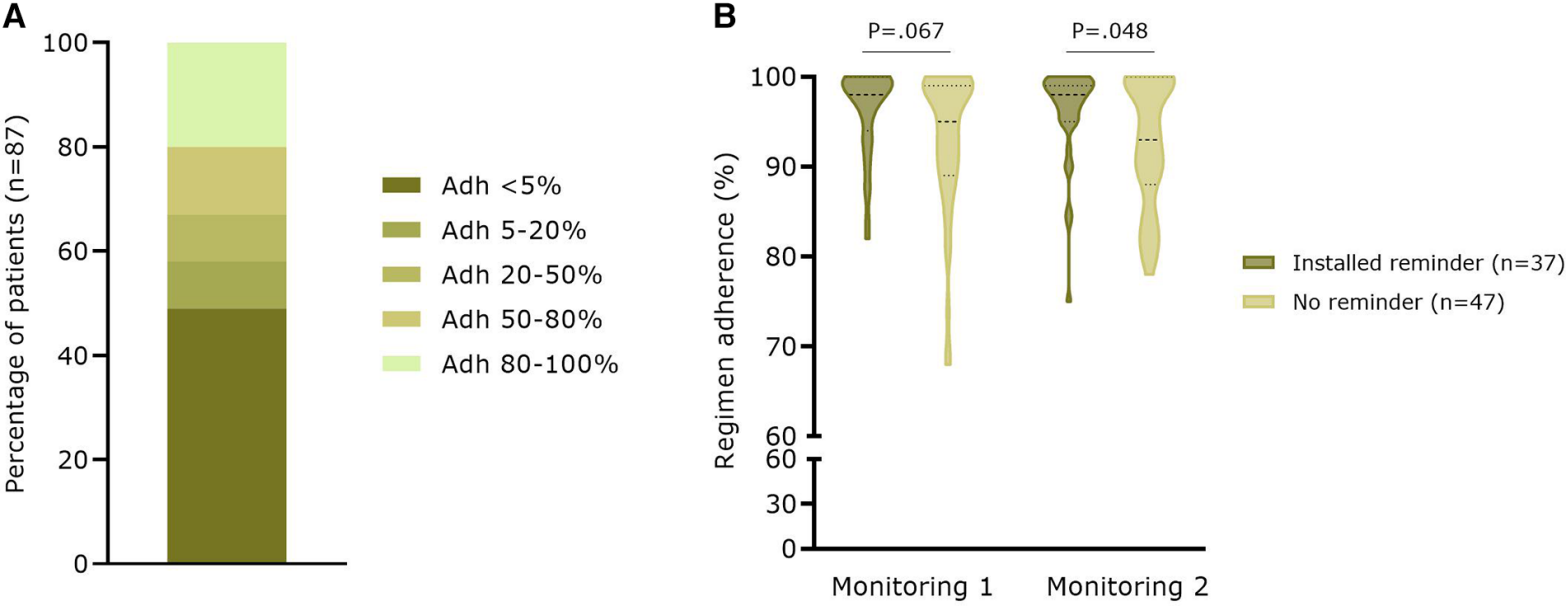
Therapy adherence based on app user data and electronic monitoring with the MEMS (**A**) Percentage of patients (*n* = 87) with different categories of therapy adherence to OAC based on indicating taken in the medication module (**B**) Real-world regimen adherence (*n* = 84) based on electronic monitoring with the MEMS. Adh, therapy adherence.

A total of 138 patients created a medication list with a median of 6.0 (3.8–10.0) medications per patient (mean: 7.2 ± 5.3 added medicines). Of these 138 patients, 103 (74.6%) effectively set at least one reminder for a drug. For a total of 87 patients who set a reminder on OAC medication, a median therapy regimen adherence (i.e., the number of days the patient recorded “medication taken” in the App, once or twice daily depending on the regimen, over the total number of days the reminder was activated) of only 5.6% (0.2–64.3%) was calculated (mean: 31.6 ± 39.2%). As shown in Figure 3A, 49% of the patients had an adherence <5% based on self-confirmed intake via the App. On the contrary, a high regimen adherence based on electronic monitoring (i.e., the proportion of days with the correct number of openings as recorded by MEMS) was seen for 84 App patients who completed two monitoring periods of 3 months at baseline and after 12 months (M1: 94.5 ± 7.0%; respectively M2: 94.1 ± 6.6%; *P* = .266). At M1, there was already a trend toward a significantly higher regimen adherence in patients with an active medication reminder in the App (96.3 ± 4.8% vs. 93.1 ± 8.0%, *n* = 84; *P* = .067). This higher regimen adherence was confirmed at M2 (96.2 ± 5.3% vs. 92.6 ± 7.1%; *P* = .048) (Figure 3B).

### 3.2. Communication

**Figure 4:**
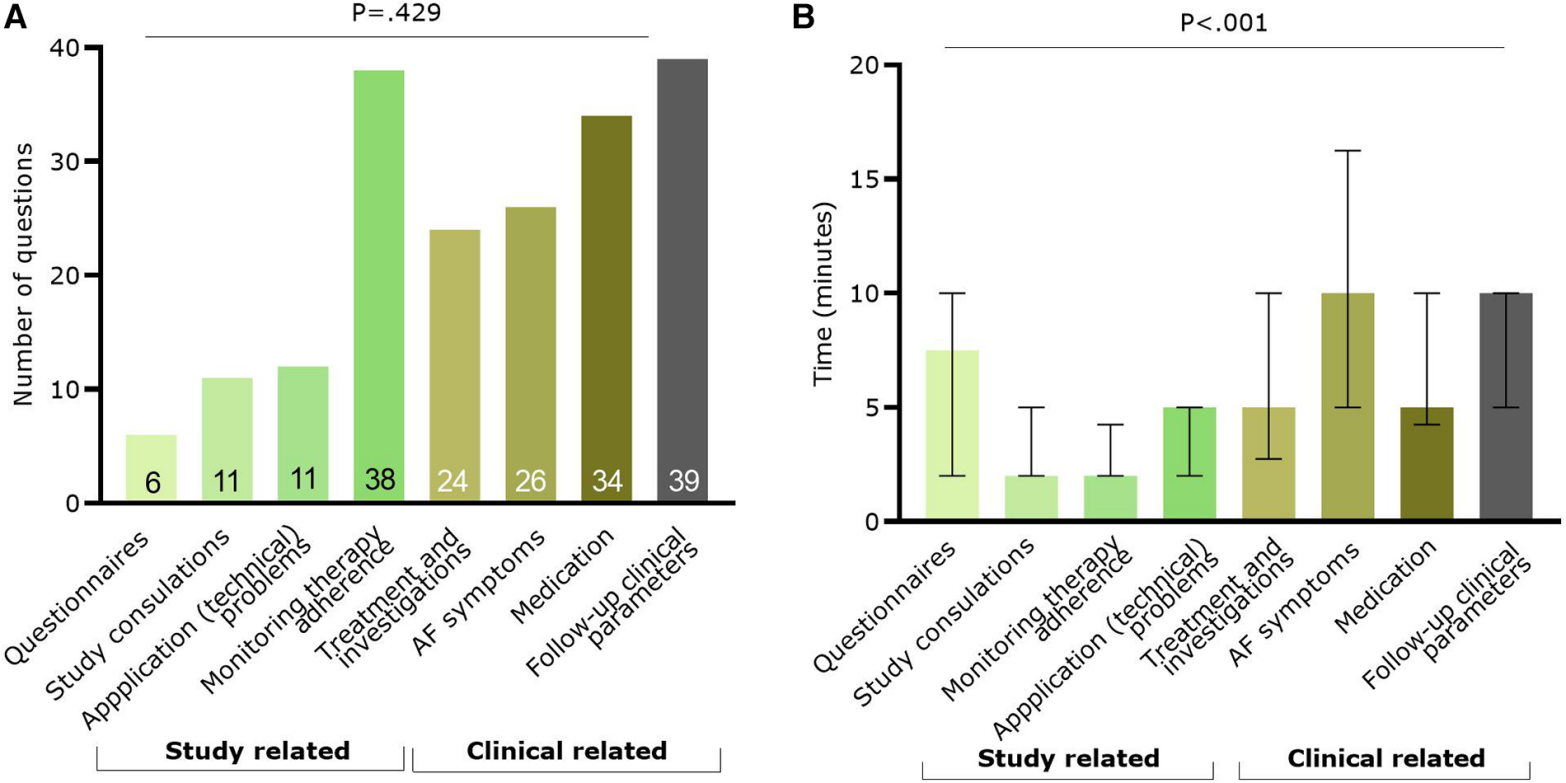
Information on the questions raised by the app patients (*n* = 75). In green the categories with study related questions and in brown/grey categories related to clinical questions. (**A**) Number of questions per classification; (**B**) Median (and IQR) time spent to answer questions per category. AF, atrial fibrillation.

A total of 190 questions were asked by 75 App patients during the study, with a mean number of 2.5 ± 2.4 questions per patient. More questions were asked online or via the application (*n* = 116; 61.1%) compared to phone contact (*n* = 74; 38.9%) in the App group. Most questions were related to recorded clinical parameters (*n* = 39; 20.5%), the application itself (*n* = 38; 20.0%), and medication (*n* = 34; 17.9%) (Figure 4A). Looking at the time necessary to answer questions, there was a significant difference between question categories (*p* < 0.001, Figure 4B). Most time was spent on questions related to AF symptoms [median: 10.0 (5.0–16.3) minutes] and on follow-up of clinical parameters [median: 10.0 (5.0–10.0) minutes].

### 3.3. Satisfaction of the AF-EduApp intervention

**Figure 5:**
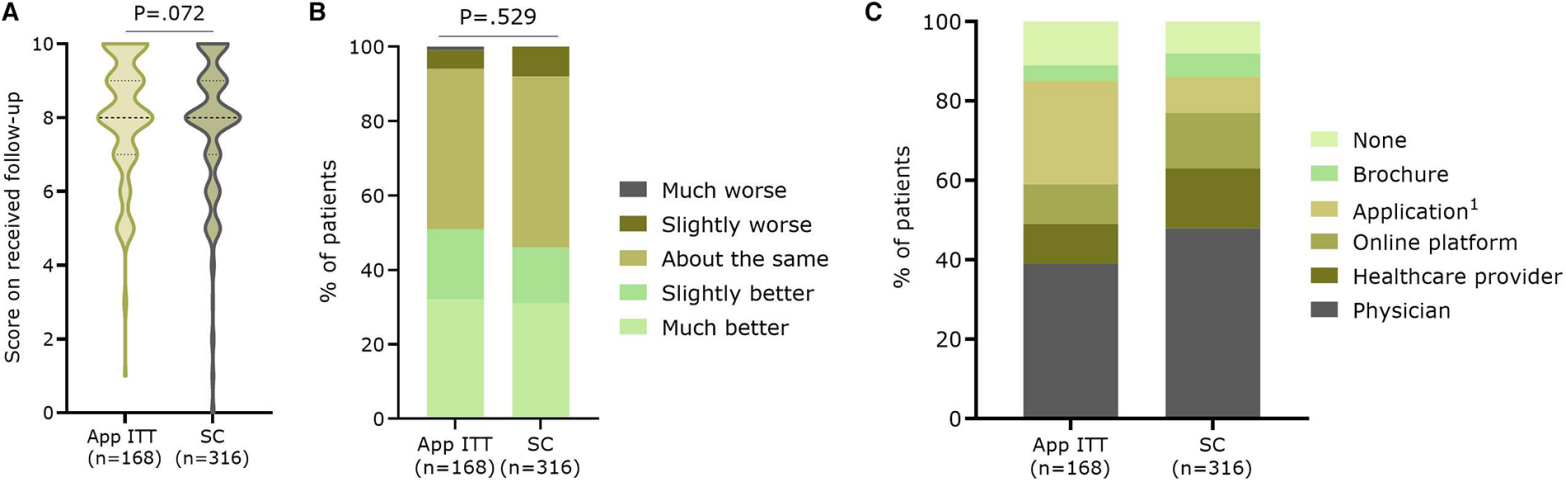
Satisfaction with the follow-up. Patients gave (**A**) a score on ten concerning their overall perceived quality of follow-up during the study, (**B**) an indication of the evolution of their health during the past year, and (**C**) which future follow-up methods they would prefer. App ITT: Application group intention-to-treat (i.e. App and no-App group); SC, Standard care group 1. *P* < .001 between App ITT and SC.

Patients gave a median score of 8.0 (IQR7.0–9.0)(mean: 7.8 ± 1.9) on ten about perceived quality of follow-up they received during the year of study, with a median score of 8.0 (IQR:7.0–9.0) in the App ITT group (*n* = 168; mean: 8.1 ± 1.7) compared to 8.0 (IQR:7.0–9.0) in the SC group (*n* = 316; mean: 7.7 ± 2.0; *P* = .072) (Figure 5A). In addition, patients could indicate how they perceived the evolution of their health over the past year. There was no significant difference between the App ITT and SC groups (*P* = .529) (Figure 5B). However, most patients considered their health the same or better. When asked which follow-up methods patients would prefer for future care, the weighted percentage showed that follow-up by a physician was most chosen in both groups (App ITT: 39.0%, SC: 47.9%; *P* = .112) (Figure 5C). Significantly more patients in the App ITT group would prefer follow-up with an application (26.1%) compared to the SC group (8.5%; *P* < .001).

**Figure 6:**
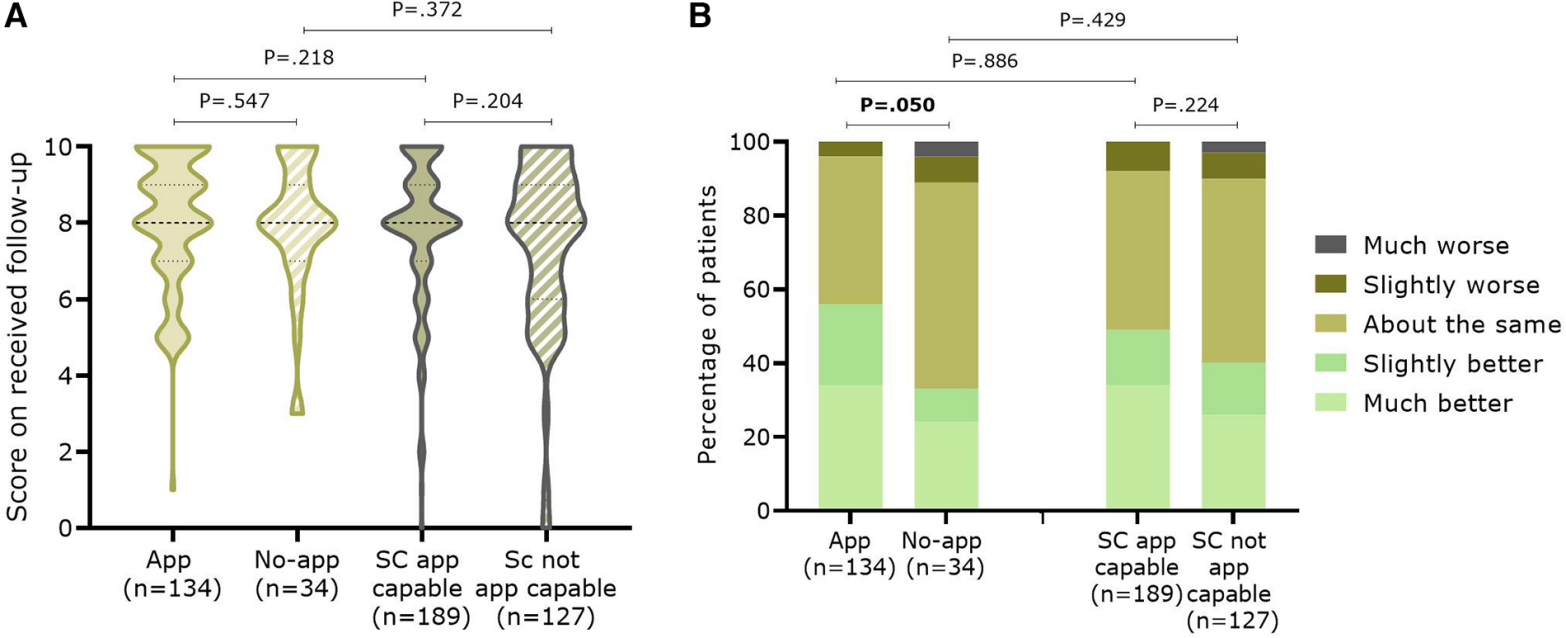
Satisfaction with the follow-up in the past twelve (App ITT) or eighteen months (SC) between patients capable or not with an application. Patients gave (**A**) a score on ten concerning their overall follow-up during the study, (**B**) an indication of the evolution of their health during the past year.

When comparing App-capable and non-capable patients within each group, no significant differences were seen within the App ITT or SC group for the perceived quality of follow-up **(**Figure 6A**)**. There was a near-significant trend for App group patients to perceive “slightly or much better health” over the last year, while most no-App patients considered their health unchanged (*P* = .05) (Figure 6B). For both App ITT and SC, significantly more patients in the non-capable App groups preferred future follow-up with a brochure with information about AF (12.3% vs. 2.2%; *P* = .003 and 9.3% vs. 4.4%;*P* = .005, respectively)**(**Table 2**)**. As expected, more patients in the App-capable groups would prefer follow-up with an application (31.6% vs. 4.4%; *P* = .001 and 12.5% vs. 2.6%; *P* < .001, respectively). Patients who were effectively followed up with the App indicated less need for follow-up with a physician (34.6% vs. 43.9%; *P* = .046) than the SC App-capable group. In addition, they indicated significantly more that they preferred follow-up with an App (31.6% vs. 12.5%; *P* < .001) compared with the SC App-capable group. Looking at the total number of patients, 56 of the 134 App patients (41.8%) prefer the use of an application as part of their follow-up compared to 42 of the 189 SC App capable patients (22.2%) (*p* < 0.001).

**Table 2:** Patients’ preferences for future follow-up methods. The different responses were considered in relation to the total number of indicated follow-up methods and presented as percentages.

	Physician	Healthcare provider^a^	Brochure	Online platform	Application	None
App	34.6*	8.7	2.2	10.6**	31.6***	12.3
(*n* = 134)						
No-App	56.4	12.7	12.3	8.3	4.4	5.9
(*n* = 34)						
*p*-value	**.** **013**	.788	**.** **003**	.876	**.** **001**	.516
SC app-capable	43.9*	12.6	4.4	18.0**	12.5***	8.6
(*n* = 189)						
SC not app-capable	53.7	17.7	9.3	8.4	2.6	8.3
(*n* = 127)						
*p*-value	.184	.194	**.** **005**	**.** **011**	**<** **.** **001**	.175

Differences between the App and SC app-capable were seen:

Bold values are significant *p*-values (i.e., *p* < 0.05).

* *p* = 0.046.

** *p* = 0.027.

*** *p* < 0.001. No significant differences were seen between the no-App and SC not app-capable group.

^a^ i.e. nurse, psychologist, social worker,…

Looking specifically at intervention satisfaction, almost three in four App patients (98/134; 73.1%) would participate again in this project in the future, and 114 (85.1%) were more motivated to be actively engaged in their health due to the extra education. Patients were satisfied with the different aspects of their follow-up as almost all patients (*n* = 129, 96.3%) found the education understandable, 120 (89.6%) reflected to have learned more about AF due to the education, and 128 (95.6%) indicated they received adequate answers on their questions However, only 93 (69.4%) found the notifications (e.g., medication reminders, questionnaires to be filled out) helpful. All aspects of the patient feedback are shown in Supplementary Figure S4.

## 4. Discussion

This is the first paper reflecting on the engagement of an in-house developed application for the management of AF, AF-EduApp. Patients did use the application one third of the time, and 81% of the patients still engaged with the application at the end of the study. Moreover, the App resulted in a low threshold for asking questions. Despite being an educational application, the Education module was the least used. Although App ITT and SC patients were satisfied with the follow-up they received the past year, a difference in future follow-up methods was seen between App and SC App-capable patients. It appears that follow-up with the App encourages further follow-up with an application and possibly decreases the need for only follow-up with a physician.

### 4.1. AF-EduApp engagement

In the literature, only a few App-based educational efforts for AF patients have been described: i.e., the UNC AF app, mAFA app, MyAF app, and the Health Buddies app (20–23). Pattern Health (Durham, NC) developed the UNC AF app to deliver AF education (including videos) over four weeks. Moreover, patients could also track parameters, medication adherence, AF symptoms, and self-reported exercise (20). A total of nine patients participated in the focus groups with the UNC AF app. In those patients, retention was 80% after one week and only 32% after four weeks. The mAFA app mainly focused on clinical decision support for physicians but also incorporated patient education and patient self-care components (21). Satisfaction was assessed for the mAFA application after one month, with 110 of the 113 patients using the application. The MyAF application is linked with a physician application to share symptoms and quality of life. In addition, this application also provides education on AF and its treatment (22). No user or usability data were described. Lastly, the Health Buddies app was based on daily challenges between the AF patient and their grandchildren to improve AF knowledge and OAC therapy adherence (23). A total of 15 patients started using the application, with a mean percentage of logged-in days of 57.7% and a significant decrease in app use by the patients after 90 days (*P* = .009). Despite these applications being studied and used, little is written about their usability and how long patients used them. During the 1-month pilot study of the AF-EduApp, a significant reduction in the odds of app use was seen from 100% of patients using the application at the beginning to 30% using the application after one month (13). These results, in combination with the results of this report, show that it is hard to motivate patients to use the application for extended periods. Within our study, a higher percentage of patients used the application during periods in which questionnaires were presented to them compared to periods without questionnaires. According to our knowledge, this is the first paper reflecting on daily use for such a long period of follow-up. Given that the patients could use the AF-EduApp according to their own needs (except when questionnaires were presented), it is encouraging to see that the App was used one third of the time for about one year.

During the focus groups with the UNC AF app, patients found it too time-consuming to input all the measurement data themselves (20). Also, during the AF-EduApp pilot study, it was suggested to couple the App to other healthcare devices to allow automatic parameter entries (13). In general, it is seen that patient adherence to these instruments decreases as the amount of work required of them increases (24). However, due to time constraints and technical issues, this was not yet implemented in the AF-EduApp. As the Measurement module was the most used during this longer follow-up study, it proves that such automatic data integration may be a priority for future development.

In contrast to the pilot study, where the Education module was the second most used module, it was now the least used module. One reason could be that patients find additional information useful, but only once or when they have a specific question (e.g., about their treatment and self-care, as this was the most visited module). Possibly a more important reason is the fact that patients were given the knowledge questionnaire (JAKQ) five times during their one-year follow-up, which forms the basis of the AF-EduCare/AF-EduApp approach (9). By increasing patients' knowledge with targeted education, we wanted to ensure that patients are more engaged in their AF care. Given that patients of the App group already had a JAKQ score of ±70% at baseline and the JAKQ was frequently given, this could provide them with the feeling that they already have enough knowledge resulting in less time spent on the Education module. Despite identical baseline knowledge (AF diagnosis <1 month: 69.6 ± 16.9% vs. AF diagnosis >1 month: 69.8 ± 14.6%; *P* = .920), it was surprising to see a significant difference in the number of days patients with recent AF diagnoses spent in the Education module compared to those with older diagnoses. However, there was no significant difference in the time spent in the Education module between patients with a recent or those with a longer AF diagnosis. This indicates a difference in the usage of the Education module between those patient groups, possibly related to time since basic AF information was given with AF diagnosis. For example, patients with a longer diagnosis appear to watch the education more often with shorter sessions spread over several days (i.e., longer time since AF basic information and need for specific information), while patients with a recent diagnosis are more likely to view education in one long episode (i.e., still need to absorb all the information).

Intriguingly, significantly more patients with a recent diagnosis discontinued the trial earlier (i.e., <12 months of follow-up) compared to patients with a longer diagnosis (*p* < 0.001). This also seems to result in fewer days with App use for this patient group. It could be that a more intensive follow-up for patients with a recent diagnosis is too overwhelming, as 27.8% of the patients quit after one month. This definitely requires further study because it is contrary to what seems medically appropriate.

As the primary goal of the educational application was to improve therapy adherence, it is positive to see that the Medication module was the second most used module, with 138 of the 152 patients creating a medication list and 74.6% of the patients setting a reminder. However, therapy adherence calculated based on patients' own recording of medication intake is embarrassingly low. Given that the actual regimen adherence as measured with electronic monitoring was very high, it seems sufficient for patients to use the medication list and reminders. Interestingly, regimen adherence was significantly higher for patients who set a reminder than those who did not.

With the usage of one third of the available time, the application use was already quite good as the patients could use the application according to their own needs. The results also showed that self-care modules, such as the measurement module, are frequently used and that interactive medication reminders positively affect therapy adherence. However, to further improve adherence to the application, one could focus more on the specific needs of patients. For example: Use interactive reminders to enter parameters in the measurement module for patients with AF symptoms or a dysregulated blood pressure; push education when starting medication or undergoing surgery; encourage to set medication reminders with a feedback loop for patients with low adherence.

### 4.2. Communication

Many questions asked during follow-up were related to the functioning of the application itself. This could indicate that, despite some difficulties, patients want to use the application. Nevertheless, it is also essential to further optimize the App based on these questions. Next, many clinical related questions were asked, requiring the most time to answer. This points to one of the main components of integrated care, i.e. a patient-centered approach with patient involvement and empowerment (25). To do this, patients must be well-educated to interact with their healthcare providers. This was, in fact, the prime goal of the AF-EduApp. Moreover, communication is crucial for patient satisfaction with their care, which was shown by others before (26, 27) and confirmed in our study. The possibility to easily ask questions can comfort patients and reduce emergency department (ED) visits or early consultations, as was shown by *Hong KL* et al. When looking at the reasons for ED attendance of AF patients in Canada, they noted that 271 of the 356 ED visits were, in fact, unnecessary and could have been managed more effectively outside the ED setting (28). Therefore, accessible communication can help patients explore whether an urgent visit is needed.

### 4.3. Satisfaction of the AF-EduApp intervention

It seems that patients of the App ITT were slightly more positive about the follow-up they received over the past year compared to the SC group (8.0 (IQR:7.0–9.0)(mean: 8.1 ± 1.7) vs. 8.0 (IQR:7.0–9.0)(mean: 7.7 ± 2.0); *P* = .072). However, no difference was seen when patients were asked how they perceived their health status, despite their health remaining the same or having improved. When asked about future follow-up, physician contact remained very important for both groups. However, more patients of the App ITT would also like future follow-up with an application compared to the SC group (*P* < .001). This was further confirmed when looking at the difference between App-capable patients from the App and SC groups versus patients who are not App-capable: more patients in the latter groups preferred follow-up with a physician or information with a brochure and less with an application.

Surprisingly, no significant differences were seen for the score on “perceived quality of follow-up in the past year” and evolution of health between the App-capable patients from the App and SC groups. Patients from the App group, who have already had a successful follow-up with an app, appear to be “ready” for more digital remote follow-up and fewer doctor visits than patients from the SC App-capable group. This indicates that experience with using an App gives patients confidence in the tool or at least did not demotivate them to use the tool, providing continuous care with targeted education, follow-up of parameters, and an easy way to ask questions if needed. However, the results on AF-EduApp engagement and the similar satisfaction rate between App-capable patients from the App and SC group show that there is still room for improvement of the application to increase app adherence and satisfaction with app-driven follow-up. Also, in the pilot study of one month, usability was positively scored: (i) 17 out of 19 patients found the application easy to use and attractive, (ii) 84.2% wanted to use the App for a longer period, and (iii) 73.1% would participate in further similar projects (13).

Similar satisfaction results were seen with other AF apps. For the mAFA trial, 93.9% of the patients found the application easy and user-friendly, 90.8% were positive on real-time communication, and 91.4% thought the application could be helpful for self-care (21). For the Health Buddies app, 9 out of 15 patients indicated they would like to use it again (23).

### 4.4. Limitations

Despite comparable demographic data between the App ITT and SC group, it should be noted that more patients in the App group had a smartphone than SC. Smartphone ownership was not a stratification factor for the randomization of the patients. However, higher smartphone ownership did not result in significant age differences, education degree or CHA_2_DS_2_-VASc score which could possibly affect patients' health. Not all available data from the AF-EduApp study arm are available for this paper. Once the complete AF-EduCare study is finished, its primary and secondary endpoints will be communicated, including those in the AF-EduApp study subgroup. This will include data on the effectiveness of the App compared to face-to-face education or an online platform on knowledge level, self-care capabilities, symptom burden, and therapy adherence.

## 5. Conclusions

Patients have a positive attitude towards using the AF-EduApp. They actively used the App for one-third of the available days and were positive about the provided follow-up and its impact on their overall health. The Measurement and Medication modules are the most used, showing patients' active involvement in their care. The App improved communication with the healthcare team. In summary, the AF-EduApp is a valid tool to supplement the care and follow-up of AF patients. Nevertheless, further initiatives can be taken to improve app adherence to increase the use of different modules such as the education module.

## Data Availability

The raw data supporting the conclusions of this article will be made available by the authors, without undue reservation.
