# Supplementary material for: Engagement of atrial fibrillation patients with the AF-EduApp, a new mobile application to support AF management
Source: Front Cardiovasc Med. 2023 Sep 26;10:1243783. doi: 10.3389/fcvm.2023.1243783 (PMC10562600; doi:10.3389/fcvm.2023.1243783)
Supplement: Supplementary file 1 [file Table1.docx]

Supplementary material: Engagement of atrial fibrillation patients with the AF-EduApp, a new mobile application to support AF management.

**Lieselotte Knaepen^1,2,3,4*^, Michiel Delesie^1,2,3^, Rik Theunis^1^, Peter Gorissen^1^, Johan Vijgen^3,4^, Paul Dendale^3,4^, Lien Desteghe^1,2,3,4^, Hein Heidbuchel^1,2,3^**

^1^Department of Cardiology, Antwerp University Hospital, Edegem, Belgium

^2^ Research Group Cardiovascular Diseases, University of Antwerp, Antwerp, Belgium,

^3^Uhasselt, Faculty of Medicine and Life Sciences, Diepenbeek, Belgium,

4 Jessa Hospital, dept. of Cardiology, Heart Centre Hasselt and dept. Jessa & Science, Hasselt, Belgium / LCRC(-MHU)

# S. Figure 1

**S. Figure 1.** AF-EduApp main screen


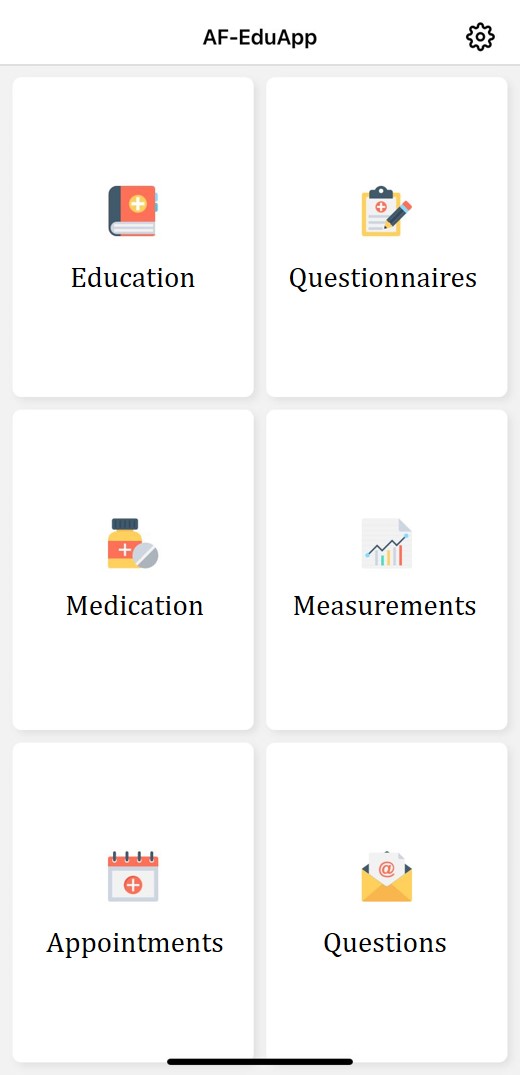


# S. Figure 2

**S. Figure 2.** Study design of the AF-EduApp study


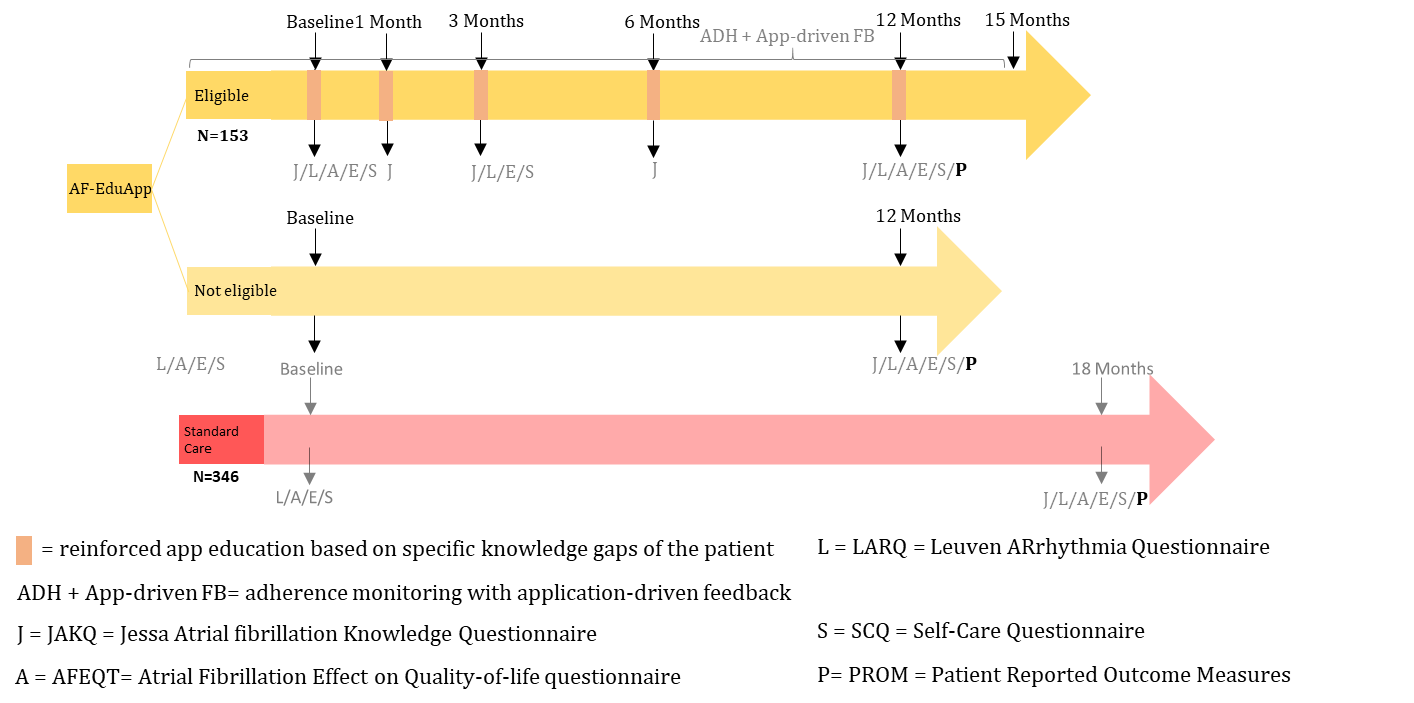


# S. Figure 3


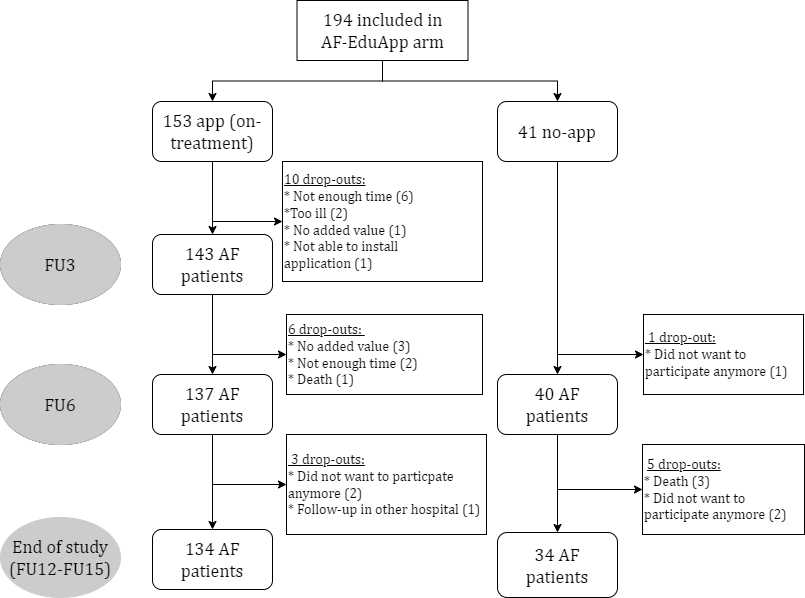


**S. Figure 3.** Flowchart follow-up of patients during 12 to 15 months

# S. Figure 4


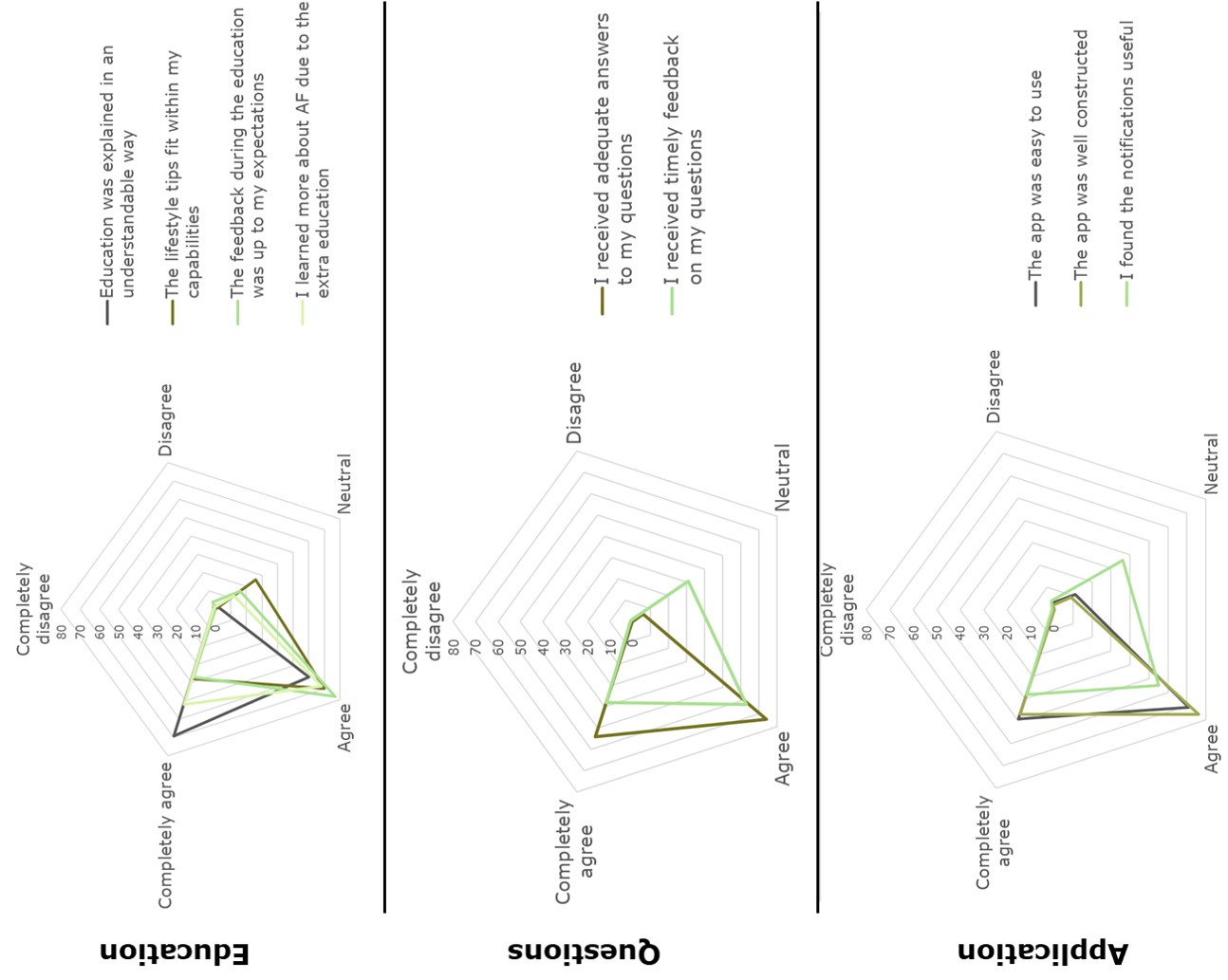


**S. Figure 4.** Satisfaction with the received follow-up shown as radar charts with number of app patients (n=134).

# S. Table 1

| **Table 1:** Demographic data of included atrial fibrillation patients | | | | |
| --- | --- | --- | --- | --- |
|  | **Total population  (n=194)** | **AF-EduApp (on-treatment)  (n=153)** | **No App  (n=41)** | **p-value^1^** |
| **Male, n (%)** | 132 (68.0%) | 105 (68.6%) | 27 (65.9%) | 0.735 |
| **Age (years), mean±SD** | 70.1 ± 7.0 | 68.8 ± 6.7 | 75.2 ± 5.6 | **<0.001** |
| **Education degree, n (%)**  Primary /secondary school  College/University | 110 (56.7%)  84 (43.3%) | 80 (52.3%)  73 (47.7%) | 30 (73.2%)  11 (26.8%) | **0.017** |
| **In possession of, n (%)**  PC/Laptop  Tablet  Smartphone | 154 (79.4)  105 (54.1)  156 (80.4) | 132 (86.3)  98 (64.1)  150 (98.0) | 22 (53.7)  7 (17.1)  6 (14.6) | **<0.001**  **<0.001**  **<0.001** |
| **Internet accessibility, n (%)** | 181 (93.3) | 153 (100.0) | 28 (68.3) | **<0.001** |
| **Time since AF diagnosis (years), mean±SD** | 5.1 ± 6.4 | 5.2 ± 6.7 | 4.4 ± 5.3 | 0.468 |
| **CHA2DS2-VASc score, mean±SD** | 3.1±1.6 | 2.9±1.4 | 4.2±1.7 | **<0.001** |
| **Anticoagulation therapy**  NOAC  VKA  LMWH  None | 161 (83.0)  16 (8.2)  0 (0.0)  15 (7.7) | 124 (81.0)  13 (8.5)  0 (0.0)  14 (9.2) | 37 (90.2)  3 (7.3)  0 (0.0)  1 (2.4) | 0.337 |
| 1. A Mann-Whitney U test was used for continuous data and a Chi-square test was used for categorical data  AF: atrial fibrillation; NOAC: Non-vitamin K antagonist Oral Anticoagulant, VKA: Vitamin K Antagonist, LMWH: Low-Molecular-Weight Heparins | | | | |
